# Follow‐up study of veterans with white and red oral mucosal lesions at Veterans Affairs Dental Clinics

**DOI:** 10.1002/cre2.677

**Published:** 2022-12-12

**Authors:** Robert S. Redman, Scott R. Diehl, Trina Jones‐Richardson, Rebeka G. Silva, Chih‐Ko Yeh, Kevin J. Malley, Sam E. Farish, Mary B. Duffy, Robert M. Craig, Deborah M. Winn

**Affiliations:** ^1^ VA Medical Center Washington District of Columbia USA; ^2^ Rutgers School of Dental Medicine Newark New Jersey USA; ^3^ VA Medical Center San Francisco California USA; ^4^ Audie L. Murphy Division South Texas Veterans Health Care System San Antonio Texas USA; ^5^ VA Medical Center Danville Illinois USA; ^6^ VA Medical Center Atlanta Georgia USA; ^7^ VA Medical Center Durham North Carolina USA; ^8^ VA Medical Center San Antonio Texas USA; ^9^ National Cancer Institute Bethesda Maryland USA

**Keywords:** leukoplakia oral, lichen planus oral, mouth neoplasms, tobacco use

## Abstract

**Objectives:**

This analysis examined the clinical and histopathological characteristics of white and red oral mucosal lesions and patient lifestyle behaviors to understand how the lesions changed over 19–23 years, including among patients who developed oral and pharyngeal cancer.

**Materials and methods:**

Seventy‐five individuals with red and/or white oral mucosal lesions with clinical diagnoses of smokeless tobacco lesions, leukoplakia, erythroplakia, lichen planus, ulcer, and virus‐associated lesions were identified in six Veterans Affairs Medical Center Dental Clinics (VAMC) from 1996 to 2001. Biopsy results and patients' sociodemographic, medical, and tobacco/alcohol use characteristics were obtained. Study dentists used standardized forms to capture information about the lesions. Study participants were re‐examined at intervals through January 2002. In 2020, a retrospective review of VAMC and public records ascertained whether participants developed oral cancer or died.

**Results:**

The most common red or white oral mucosal lesions among the 75 study participants were leukoplakia (36.0%), smokeless tobacco lesions (26.7%), virus‐associated lesions (18.7%), and lichen planus (16.0%). Lesions in 11% of participants with leukoplakia and one‐third of participants with lichen planus persisted for 5 years or more. Dysplasia was present in four participants with leukoplakia. Seventeen percent of participants developed a new white or red oral mucosal lesion. Five patients (6.1%) developed oral or pharyngeal cancer, four among participants with leukoplakia (one with prior dysplasia) and one among participants with lichen planus. Four of the cancers developed 6–20 years after enrollment, and only one was at the original lesion site.

**Conclusions:**

The occurrence of oral and pharyngeal cancers in some study participants with white and red oral mucosal lesions many years after enrollment reinforces the need for patients, dentists, and health care systems to have better methods to identify and assess the malignant potential of oral lesions, monitor patients over time, and intercept high‐risk oral lesions before they become cancerous.

## 1 INTRODUCTION

White and red oral mucosal lesions are relatively common in adults (Shulman et al., 2004), and some may have malignant potential and progress to malignancy (Warnakulasuriya et al., 2021). However, very few of those lesions will become cancerous. Dental providers encountering these oral lesions must decide how to clinically characterize them, whether to biopsy patients, where in the oral cavity to biopsy, and how often to monitor the lesions. This is challenging because of the many possible diagnoses of clinical oral lesions (Nelson & Thompson, 2019), potentially modifiable risk factors causing lesions such as tobacco use (Porter et al., 2018), limited accuracy of less invasive diagnostic tests (Walsh et al., 2021), limited treatment options beyond lesion excision (Lodi et al., 2016), and an absence of good biomarkers of lesion progression (Lingen et al., 2017). If oral mucosal lesions with the greatest potential for malignant transformation can be identified, dentists could better monitor the lesions and treat them to intercept the development of oral cancers.

Evidence at the time that the study was initiated suggested that oral cancers may show genetic changes; for example, p53 mutations were noted in more than a third of head and neck cancers (Greenblatt et al., 1994) and were found in some leukoplakias as well (Girod et al., 1994; Lazarus et al., 1995) suggesting that genetic changes could be markers of the risk of malignancy. The study aimed to examine candidate gene mutations and other emerging genetic factors as well as tobacco use among persons with red and white oral mucosal lesions warranting biopsy such as lesions with known malignant potential (e.g., lichen planus). The secondary aims of the study were to characterize changes over time in the clinical and histopathologic features of the lesions and in relation to candidate genetic markers and behaviors such as tobacco use and to create a data and biospecimen repository to enable future studies.

This study was initiated at dental clinics in Veterans Affairs Medical Centers (VAMCs) and involved collecting clinical, histopathologic, and behavioral data about the lesions and the patients using standardized forms and procedures. Although study accrual ended without reaching the expected sample size, this study population, with 2 decades of follow‐up, provides valuable information about the long‐term behavior of oral lesions and participants who developed oral or pharyngeal cancer.

## 2 METHODS

Patients in whom red or white oral mucosal lesions were identified at Dental Clinics at six United States Department of VAMCs between October 1996 and January 2002 were enrolled in the study. These were Washington, DC; Danville, Illinois; Durham, North Carolina; Atlanta, Georgia; San Francisco, California; and San Antonio, Texas VAMCs. This study was approved by the Institutional Review Board of the National Institute of Dental Research and by the Human Subject and Research and Development Committees of each VAMC and conforms to the Strengthening the Reporting of Observational Studies in Epidemiology guidelines. Types of lesions eligible for study enrollment were those that, when the study was initiated, had been associated with an elevated risk of malignant transformation in other studies (e.g., leukoplakia) or as markers for persons at high‐risk of oral cancer (e.g., hairy leukoplakia). A previous report described findings related to study participant self‐recognition of the oral lesions (Shugars et al., 2007).

When a dentist found a red or white oral mucosal lesion during routine outpatient examinations and care, ruled out obvious causes such as denture frictional lesions, and the standard of care for the lesion was biopsy, the person was invited to enroll in the study. Study dentists were calibrated to the clinical diagnoses via diagnostic criteria with presentations and tests using color slides of eligible and similar‐looking ineligible lesions. Eligible oral mucosal lesion clinical diagnoses were smokeless tobacco lesions (which required a smokeless tobacco use history), leukoplakias, erythroplakias, lichen planus, virus‐associated lesions (papilloma, verruca vulgaris, condyloma acuminatum, and hairy leukoplakia), Kaposi's sarcoma (no study participants had this diagnosis), and red/white ulcer, as described in Appendix Table A1 (Westat Inc, 1992). The locations and other clinical features of each oral lesion were recorded on a standardized form. Multifocal and clustered individual lesions were distinguished from isolated single lesions and diffuse lesions that covered a large area of the mouth (i.e., generalized). Persons with previously treated cancers of the oral cavity, or other head and neck cancers previously treated by therapeutic irradiation, were excluded from the study. Although the target sample was 250 patients, enrollment was terminated at 79 patients because of slow accrual due to changes in Department of Veterans Affairs (VA) dental eligibility policies that resulted in net fewer patients being seen in the dental clinics.

After the study was described to the patients with eligible lesions and written informed consent was obtained, study participants received an intraoral examination to identify and characterize the oral lesions; lesions were photographed; oral epithelial cell brushing samples were taken from the lesion and a normal oral mucosal site for later genetic analysis; a biopsy was performed and a small piece of the specimen not needed for diagnostic purposes was stored for genetic analyses; and the study participants were interviewed using a standardized questionnaire for information about sociodemographic, medical, and tobacco use and alcohol‐drinking behaviors. Histopathologic diagnoses were established in the respective VAMCs.

Between October 1996 and January 31, 2002, study participants returned every 4–6 months or at the professional discretion of the study dentist. At every visit after enrollment, study participants were examined using the same protocol as for the enrolling visit. This time period involving repeat dental examinations will be referred to as the prospective follow‐up.

A retrospective, chart review was undertaken in 2020 and will be referred to as the retrospective, long‐term follow‐up. All study participants were followed up through 2020 to determine the presence or new occurrence of white and red oral mucosal lesions, oral cancer diagnoses, and deaths and cause of death through a review of VA dental and medical records across the VAMC system. For participants with no evidence of death in VA records, abstractors determined the participants' date last known alive based on their health record indicating that they had interacted with the VAMC, such as a dental or medical visit, hospitalization, telephone call with, or mail from the participant. In addition, abstractors searched the internet for an obituary or other notice of death. Since the earliest enrollments occurred in October of 1996 and the last ones in March 2001, the entire follow‐up period was 19 to 23 years. The abstractors were the original study dentists (or their successors) at the VA dental clinics with one original dentist overseeing the effort.

Users of various forms of tobacco were persons who had ever smoked 100 cigarettes, cigars, or pipes or who had used snuff or chewing tobacco for 6 months or more. Tobacco users were categorized as current or former users based on their response to questions about use of the products “now.” Pack‐years of cigarette smoking was defined as the number of packs per day of cigarettes that the study participant usually smoked times the number of years that the person smoked cigarettes. Alcohol users had had at least 12 alcoholic beverage drinks of any type (e.g., beer, hard liquor) in their lifetime and were also classified as never, current, and former users. A *χ* ^2^ test of association was used to evaluate whether the clinical diagnosis (e.g., lichen planus) was independent of observable clinical features (e.g., lesion size), histopathologic features, and tobacco and alcohol use characteristics. If the *p* ≤ .05, the null hypothesis of independence is rejected, meaning that the feature/characteristic and clinical diagnosis categories are associated with each other (i.e., the frequency distributions of the feature/characteristic are not the same across the clinical diagnosis categories). The *z*‐score test for two population proportions was used to evaluate differences between two groups in the proportions developing cancer.

## 3 RESULTS

Seventy‐nine participants were enrolled in the cohort. Four study participants with red or white mucosal lesions were diagnosed histopathologically with in situ or invasive oral squamous cell oral cancer at the time of or within a month of the enrolling visit and were excluded from further analysis, leaving 75 participants, only one female, in this analysis. Fifty‐five (73.3%) were White; 16 (21.3%) were Black; two (2.7%) reported more than one race; one (1.3%) was American Indian/Alaskan Native; and one (1.3%) was Asian or Pacific Islander. Four persons were of Hispanic origin. The age range was 27–78 years, and the median age was 58 years. The number of participants enrolled at the six VA Medical Centers varied from 3 to 20.

Among the 75 participants, the first listed clinical diagnoses of red and white mucosal lesions were leukoplakia (27 participants [21 with homogeneous and 6 with nonhomogeneous leukoplakia], 36.0% of the participant population), smokeless tobacco lesions (20 participants, 26.7%), virus‐associated lesions (papilloma, verrucous vulgaris, and condyloma acuminatum; 14 participants, 18.7%), and lichen planus (12 participants, 16.0%). One participant with an erythroplakia and another with a nonspecific ulcer are included in the Tables' summary columns.

Table 1 describes the key features of the different clinically diagnosed lesions. More than a quarter of the participants (26.7%) had more than one white or red mucosal lesion at the enrolling visit. Of the 95 lesions, 61.9% were single, defined lesions (vs. multifocal or generalized lesions) and 71.8% were white. Participants had been aware of a third of the lesions (32.0%) for more than 2 years. Dentists had previously detected 73.2% of the lesions, two thirds within the past 6 months. Most biopsies were incisional (55.7%). Only 12.4% of the lesions were painful. Some lesion characteristics were associated with the clinical diagnosis categories of smokeless tobacco lesions, leukoplakia, lichen planus, and virus‐associated lesions based on the *χ* ^2^ test for association. The characteristics that varied across clinical diagnostic categories included the pattern of lesions (i.e., single, multifocal, generalized); the longest dimension of single lesions; lesion color; how long the participant was aware of the lesion; biopsy type (incisional/excisional); and histopathologic features.

**Table 1:** Clinical characteristics of lesions present at enrollment by the first listed clinical diagnosis

	Clinical diagnosis									
Lesion characteristic	Smokeless tobacco lesions		Leukoplakia		Lichen planus		Virus‐associated lesions		Total^a^	
	#	*%*	#	%	#	%	#	%	#	% (*p* Value)
# Study participants	20		27		12		14		75	
# Lesions at enrolling dental visit										
1	16	80	16	59.3	10	83.3	11	78.6	55	73.3
2 or 3	4	20	11^b^	40.7	2^c^	16.7	3^d^	21.4	20	26.7 (0.27)
Total # lesions	24		38		16		17		97	
Pattern of lesions										
Single	15	62.5	23	60.5	7	43.8	14	82.4	60	61.9
Multifocal	0	0	6	15.8	5	31.3	3	17.6	14	14.4
Generalized	9	37.5	9	23.7	4	25.0	0	0	23	23.7 (0.02)
Longest dimension of single lesions										
1–9mm	3	20.0	8	34.8	1	14.3	13	92.9	25	41.7
10–19 mm	2	13.3	10	43.5	4	57.1	1	7.1	18	30.0
≥20	10	66.7	5	21.7	2	28.6	0	0	17	28.3 (0.000)
Lesion colors										
White	15	62.5	34	89.5	8	50.0	12	70.6	69	71.1
Red	1	4.2	0	0	0	0	0	0	2	2.1
White and red	4	16.7	1	2.6	8	50.0	2	11.8	16	16.5
Gray	3	12.5	1	2.6	0	0	0	0	4	4.1
Pink	0	0	0	0	0	0	2	11.8	2	2.1
White and gray	0	0	2	5.3	0	0	0	0	2	2.1
Normal	1	4.2	0	0	0	0	0	0	1	1.0
Missing	0	0	0	0	0	0	1	5.9	1	1.0 (0.001)
Participant‐reported duration of lesion										
Unaware of any lesions	4	16.7	22	57.9	6	37.5	6	35.3	39	40.2
≤1 month	0	0	2	5.3	1	6.3	0	0	3	3.1
1–<6 months	1	4.2	5	13.2	2	12.5	4	23.5	13	13.4
6–12 months	1	4.2	0	0.0	2	12.5	1	5.9	4	4.1
>12 months–2 years	1	4.2	1	2.6	1	6.3	3	17.6	6	6.2
>2 years	16	66.7	8	21.1	4	25.0	3	17.6	31	32.0
Missing	1	4.2	0	0	0	0	0	0	1	1.0 (0.01)
Noted by dentist at previous visit										
No	3	12.5	9	23.7	2	12.5	5	29.4	20	20.6
Yes	20	83.3	27	71.1	11	68.8	12	70.6	71	73.2
Missing	1	4.2	2	5.3	3	18.8	0	0	6	6.2 (0.25)
How long since lesion was first noted by the dentist										
<1 month	4	20.0	13	48.1	3	27.3	4	33.3	25	35.2
1–<6 months	5	25.0	12	44.4	2	18.2	3	25.0	22	31.0
6–12 months	1	5.0	0	0	1	9.1	1	8.3	3	4.2
>12 months–2 years	2	10.0	1	3.7	1	9.1	1	8.3	5	7.0
>2 years	3	15.0	0	0	1	9.1	1	8.3	5	7.0
Missing	5	25.0	1	3.7	3	27.3	2	16.7	11	15.5 (0.34)
Biopsy type										
Incisional	20	83.3	21	55.3	11	68.8	1	5.9	54	55.7
Excisional	2	8.3	13	34.2	4	25.0	14	82.4	34	35.1
No biopsy	1	4.2	3	7.9	0	0	0	0	4	4.1
Missing and dk	1	4.2	1	2.6	1	6.3	2	11.8	5	5.2 (0.000)
Participants' pathologic diagnoses										
Hyperkeratosis and/or parakeratosis	15	62.5	9	26.5	2	15.4	0	0	26	29.2
Hyperplasia with or without hyperkeratosis, parakeratosis	3	12.5	11	32.4	1	7.7	4	25.0	20	22.5
Dysplasia incl. with hyperplasia, parakeratosis	0	0	4	11.8	0	0	0	0	4	4.5
Lichen planus incl. with hyperkeratosis and parakeratosis	0	0	2	5.9	6	46.2	0	0	8	9.0
Papilloma incl. with hyperplasia, parakeratosis, condyloma acuminatum and verruca vulgaris, hairy cell leukoplakia	0	0	1	2.9	0	0	11	68.8	12	13.5
Normal tissue/no atypia	3	12.5	3	8.8	0	0	0	0	6	6.7
No biopsy or path report unavailable	3	12.5	4	11.7	4	30.8	1	6.3	13	14.6 (0.000)

^a^ Includes one participant with erythroplakia and one with unspecified ulcer.

^b^ One participant had three homogeneous leukoplakias, one participant with a nonhomogeneous leukoplakia also had a homogeneous leukoplakia, and one with a nonhomogeneous leukoplakia also had a lichen planus lesion listed in the lichen planus column.

^c^ One participant had a third lichen planus lesion, and one lichen planus lesion was in a participant whose first listed lesion was a nonhomogeneous leukoplakia.

^d^ One participant with a papilloma also had a hairy leukoplakia, the only one in the study.

For the smokeless tobacco lesions, the anatomic sites most often involved were the lower vestibule, labial or buccal mucosa, and the gingiva, anatomic sites where chewing tobacco and snuff are commonly placed. Nineteen (79.2%) of the 24 smokeless tobacco lesions were 20 mm or longer in the longest dimension or described as generalized. The histopathologic diagnosis for almost two‐thirds of these lesions (62.5%) was hyperkeratosis.

Thirty‐four (89.5%) of the 38 leukoplakia lesions involved the buccal mucosa or gingival‐related sites. The other four lesions were in the soft palate (two participants), the floor of the mouth, and the tongue; none of these four participants developed oral cancer subsequently. The histopathological diagnosis for four participants with lesions was dysplasia. Other diagnoses were hyperkeratosis and/or parakeratosis without hyperplasia for 26.5% of the lesions and 32.4% were hyperplasia with or without hyperkeratosis or parakeratosis.

Lichen planus was mentioned in the histopathologic reports for half of the clinically defined lichen planus lesions, with other lesions described as hyperkeratosis, parakeratosis, or hyperplasia. Two‐thirds of the lesions included the buccal mucosa. Of the virus‐associated lesions, 82.4% were single, isolated lesions. Two‐thirds of those lesions (68.5%) had a histopathologic diagnosis of papilloma, verrucous vulgaris, or condyloma.

Tobacco and alcohol use at enrollment is shown in Appendix Tables A2a and A2b, respectively. Seventy‐seven percent of the participants were current or former cigarette smokers, and 42.7% were current or former smokeless tobacco users. The distributions of cigarette smoking and smokeless tobacco use classified as never, former, or current (*p* = .0004) and by pack‐years (*p* = .04) were not similar across (i.e., were associated with) the clinical lesion diagnosis categories of smokeless tobacco lesions, leukoplakia, lichen planus, and virus‐associated lesions. All participants with smokeless tobacco lesions currently or previously used chewing tobacco or snuff, a criterion for the clinical diagnosis. Alcohol use (current vs. never/former) was not associated with the clinical diagnosis types.

Of the 75 participants, 21.3% did not return for any prospective follow‐up visits, and 78.7% returned for between 1 and 9 dental visits. Among those who returned, the median time between visits was 5.7 months, and the median duration of follow‐up between enrollment and January 31, 2002 was 1.9 years. Nearly two thirds, 65.3%, of the participants had died through 2020. Indicators suggest that participants remained in the VA health system throughout their lives. Of the 49 who died, two thirds, 67.3%, either died in or interacted with a VAMC in the 3 years before death. No deaths were found from the internet search for obituaries for the 26 not known to be deceased, and 88.5% of them last interacted with the VA health care system as recently as 2019 or 2020.

The duration and long‐term outcomes of the lesions are shown in Table 2. Four participants had no VA dental visits in the prospective and long‐term follow‐up periods. One quarter (25.4%) of the participants no longer had a lesion present 3 or more years after the last visit when the lesion was present. Some participants (22.5%) developed new white or red oral mucosal lesions. Only 11.1% of participants with leukoplakia developed new lesions, while about a quarter to a third of the participants with other clinical diagnoses developed new lesions. More than half (53.3%) of the lesions were present for 1 year or more after study enrollment. One‐third of the participants with lichen planus had lesions present for 5 years or more.

**Table 2:** Events among participants with white and red oral lesions during follow‐up from enrollment through 2020

	Clinical diagnosis									
	Smokeless tobacco lesions		Leukoplakia		Lichen planus		Virus‐ associated lesions		Total^a^	
Type of event	#	%	#	%	#	%	#	%	#	% (*p* Value)
# Participants	20		27		12		14		75	100
Had ≥1 dental visits	19		27		11		13		71	
Had a lesion absent ≥3 years after the dentist last documented^b^	5	26.3	7	25.9	2	18.2	4	30.8	18	25.4
Developed ≥1 new white or red lesion	6	31.6	3	11.1	3^c^	27.3	4^d^	30.8	16	22.5
Duration for which at least one lesion persisted										
<1 year	6	30.0	14	51.9	3	25.0	11	78.6	35	46.7
1–4	13	65.0	10	37.0	5	41.7	3	21.4	32	42.6
5–9	0	0	3	11.1	1	8.3	0	0	4	5.3
10–14	1	5.0	0	0	0	0	0	0	1	1.3
≥15	0	0	0	0	3	25.0	0	0	3	4.0 (0.002)
# Developing oral or oro‐pharyngeal cancer	0	0	4	14.8	1	8.3	0	0	5	100 (0.16)
By cigarette smoking status at enrollment (# with cancer/# participants)										
Current	0/1		3/15		0/2		0/6		3/25	
Former	0/9		0/10		1/9		0/4		1/33	
Never	0/10		1/2		0/1		0/4		1/17	
Participant deceased										
Deceased	10	50.0	19	70.4	9	75.0	10	71.4	49	65.3
Died of oral or oro‐pharyngeal cancer	0		1	3.7	0		0		1	1.3
Presumed alive	10	50.0	8	29.6	3	25.0	4	28.6	26	33.7

^a^ Includes one participant with erythroplakia and one with unspecified ulcer.

^b^ Fully regressed or was removed and did not return.

^c^ Two participants developed 2 s new lesions.

^d^ One participant developed four new lesions.

Five participants developed oral cancer, all of them squamous cell carcinomas, through 2020. Of the study participants with oral leukoplakia, four (14.8%) developed oral, oropharyngeal, or hypopharyngeal cancer, none at the original, incisionally biopsied anatomic sites of their enrollment lesions. The proportions of persons with leukoplakia developing oral or pharyngeal cancers were not statistically significantly different between the current cigarette smokers (3 of 15) and former/never smokers (1 of 12) (*p* = .40) or between the current drinkers (2 of 11) and the former/never alcohol drinkers (2 of 16) (*p* = .41). One of the 12 study participants with lichen planus (8.3%) developed a squamous cell carcinoma at the site of one of the participant's two lichen planus lesions at enrollment 20 years later. The periods between enrollment and oral or pharyngeal cancer diagnosis for the other four patients were 1.5, 6, 8, and 14 years.

## 4 DISCUSSION

This study enrolled and followed up to 23 years persons attending VA dental clinics with white and red mucosal lesions clinically diagnosed as smokeless tobacco lesions, leukoplakia, erythroplakia, lichen planus, unspecified ulcer, and papilloma/verrucous vulgaris/condyloma acuminatum. While some lesions resolved or were surgically excised and did not recur, some persisted for years. One fifth of the study participants developed new lesions. Most red and white oral mucosal lesions do not progress to cancer (Warnakulasuriya & Ariyawardana, 2016). However, in this study, five of the 75 study participants (6.7%) developed oral or pharyngeal cancer during the two‐decade follow‐up, more than two orders of magnitude higher than the U.S. general population incidence of 15.7 per 100,000 population (i.e., 0.016%) (Surveillance, Epidemiology, and End Results (SEER) Program 2020). Four of the cancers developed in individuals who had oral leukoplakia at enrollment; thus, cancer developed in 14.8% of the individuals with oral leukoplakia between 1.5 and 18 years after enrollment in the study.

Leukoplakia (Warnakulasuriya & Ariyawardana, 2016), erythroplakia (Warnakulasuriya et al., 2021), and lichen planus (Aghbari et al., 2017) are considered to have malignant potential because persons with these lesions are at higher risk of developing oral cancer than the general population or persons without these lesions. In one large well‐conducted observational study of persons with leukoplakia, the cumulative incidence of oral cancer over 5 years was 2.5% (Yanik et al., 2015), and in another study, the 5‐year cumulative incidence of oral cavity cancer was 3.3% (Chaturvedi et al., 2020) compared to an estimated 3.7% 5‐year cumulative incidence in this study. Almost 1% of patients with oral lichen planus and oral lichenoid lesions developed oral cancer in another large study (Aghbari et al., 2017), ∼4.3% over 5 years, higher than this study's estimated 2.1% 5‐year cumulative incidence. Thus, the estimated 5‐year transformation rates in this study are generally in line with other studies. In the present study, only one cancer developed after a dysplasia diagnosis (in an oral leukoplakia lesion).

Only one of the five cancers developed at the site of the original lesion at enrollment (in an individual with lichen planus). This finding is consistent with the concept of field cancerization: “the development of an abnormal area of epithelium in which cells share a collection of early changes in the process of transformation towards malignancy” (Frampton & King, 2013). Other lines of evidence support the hypothesis that field cancerization may occur in multiple locations in oral mucosal tissues, and the oral lesion is a marker of this process. In a retrospective analysis (Chaturvedi et al., 2020), 40% of oral cancers developed among biopsied leukoplakia patients who did not have dysplasia. Also, there is an absence of reduced risk of malignant transformation after laser ablation of oral lichen planus lesions (de Pauli Paglioni et al., 2020).

In this study, no participants with smokeless tobacco lesions (who all were current or previous users of snuff or chewing tobacco) or with clinical diagnoses of virally induced oral lesions developed oral or oropharyngeal cancer. Smokeless tobacco use is a risk factor for oral cancer (IARC Working Group on the Evaluation of Carcinogenic Risks to Humans, 2012). Lesions in smokeless tobacco users have distinct clinical features (e.g., white or grayish color and corrugated texture); persistent ones are now considered to be leukoplakias (Warnakulasuriya et al., 2021). Studies are needed to better understand the malignant potential of smokeless tobacco lesions (Warnakulasuriya et al., 2021); hairy leukoplakia, which is caused by the Epstein‐Barr virus (Guidry et al., 2019); and oral papillomas, verruca vulgaris, and condyloma lesions (Syrjänen, 2018), which are caused by human papillomavirus (HPV) genotypes 6 and 11, are types considered to have low malignant potential (Pringle, 2014).

Given the evidence for field cancerization, dentists face difficulties in identifying those with lesions who are at the greatest risk of developing oral cancer and how to monitor patients with lesions over extended time periods. For example, in this study, a quarter of the lichen planus lesions found at study enrollment persisted for 15 or more years. No biomarkers of prognosis of oral leukoplakia are considered well validated (Villa et al., 2019). Moreover, the clinical decision to biopsy an oral lesion has low sensitivity and specificity and only moderate positive predictive value for detecting a prevalent or incident oral cancer compared to oral lesions not biopsied (Chaturvedi et al., 2020).

Current approaches for early detection of oral cancer in people with oral lesions include clinical staining solutions, oral cytology that involves a scraping instrument or a brush to capture cells, and light‐based detection approaches (Patton et al., 2008; Walsh et al., 2021). However, the quality of the studies and the studies of the sensitivity and specificity of these approaches versus biopsy are inadequate to recommend these approaches for early detection of oral cancer (Walsh et al., 2021). Promising noninvasive approaches, such as salivary rinses (Schussel et al., 2013) and optical mapping scopes (National Institutes of Health RePORTER & Richards‐Kortum, 2021), may help identify high‐risk lesions and areas across the whole oral mucosal field that may harbor a malignancy. There are also uncertainties around the effectiveness of treatments for patients who have oral mucosal lesions with malignant potential (Arduino et al., 2021; Lodi et al., 2016). Approaches are being developed to treat persons with oral premalignancies with medications such as metformin (Gutkind et al., 2021) that may intercept the carcinogenic process and reduce the likelihood of malignant transformation.

Tobacco smoking is a risk factor for leukoplakia (Porter et al., 2018) and for progression of lichen planus to oral cancer (Aghbari et al., 2017). Nearly all the participants in the present study with those lesions were current or former cigarette smokers. To prevent oral cancers, motivating smokers and smokeless tobacco users to quit is important, and effective behavioral risk factor interventions for dental settings are available (American Dental Association, 2021; Centers for Disease Control and Prevention, 2021).

Little is known about how patients in community settings with leukoplakia and other types of oral lesions are monitored (Thompson et al., 2022). Investigators surveyed dentists about 2 decades after community dentists sent biopsies of oral lesions in 2000 and 2001 to the University of Minnesota for histopathological analysis that were found to have dysplasia. Responding dentists conducted regular follow‐up of three‐fourths of the patients, and 11% of those patients developed oral cancer. However, only 41% of the dentists contacted responded to the questionnaire, and the status of the patients of the non‐responding dentists is unknown (Thompson et al., 2022). The investigators noted the unmet need for oral cancer surveillance and follow‐up care of these dysplasia patients and their concern about surveillance and follow‐up care in population subgroups with limited access to health care, such as those who do not have regular dental care or health insurance.

The long follow‐up of study participants is a strength of this study. Other strengths are that veterans who are eligible for medical care at VA facilities obtain free medical care throughout their lives, and most study participants were known to have remained in the VA health care system during the long‐term follow‐up. The study also afforded a unique opportunity to identify new or persistent lesions for many years even if participants moved and obtained care at a different VAMC. Oral and pharyngeal cancer diagnoses, deaths at any VAMC facility, or the date the participant was last known alive were available, though some oral or oropharyngeal cancers or deaths outside the VA system could have been missed.

Another strength of this study is the use of standardized procedures and calibration of the participating dentists. Publications (Nelson & Thompson, 2019; Thompson et al., 2022) have noted the importance of capturing clinical features of oral lesions and better integrating that information with histopathologic findings to help identify patients at highest risk of developing oral cancer. However, dentists need tools to enable them to provide clinical information about oral lesions in a standard, consistent way. Dental public health and dental provider organizations could promote the development of a consensus on standardized descriptions of clinical features of oral lesions such as size, color, and anatomic location; train dentists to use the tools; and help get these tools incorporated into dental practice. This might improve clinical management of lesions, especially when patients switch dentists, and improve recognition of changes over time in clinical features, recurrences, and lesion regression or progression. Standardized documentation protocols could also include triggers for referral and biopsies with concerning high‐risk lesion features such as large lesion size (Holmstrup et al., 2006) and red and mixed white and red colors (Jayasooriya et al., 2020).

Limitations of this study include a sample size that is not large enough to address determinants of oral cancer risk or to be generalizable to the VA or general population of adults. The findings likely reflect the experience of adult males who have dental insurance. Some participants were prevalent cases (already known to have an oral lesion at the time of enrollment). The dentists' decision to biopsy might have been influenced by the lesions' size, appearance, or persistence or to make a differential diagnosis. Some participants could have received dental care outside of the VA system and VA dentists may have overlooked the presence or absence of soft tissue lesions after the repeat dental exams in the prospective follow‐up ended, so reporting of oral lesions is probably incomplete. Finally, HPV infection is an important cause of oropharyngeal cancer, but no tests were performed to assess HPV using serology or in oral or pharyngeal tissues.

The findings of this follow‐up study for over 2 decades are consistent with and reinforce findings from larger studies that oral leukoplakia and lichen planus have the potential for malignant transformation. The development of oral and pharyngeal cancer many years after the oral lesions were identified suggests the need for long‐term monitoring of patients with white and red oral mucosal lesions, which is challenging, given lack of access to dental care experienced by many (Quiñonez et al., 2022). Research continues to be needed to identify approaches to prevent these lesions with risk factor modification, identify people and lesions at greatest risk of progression, and treat people with these lesions to intercept the risk of oral cancer.

## AUTHOR CONTRIBUTIONS

Deborah M. Winn and Robert S. Redman contributed to the conception, design, data acquisition, analysis, and interpretation of the study, and drafted and critically revised the manuscript. Scott R. Diehl contributed to the conception, design, data acquisition, analysis, and interpretation of the study, and critically revised the manuscript. Trina Jones‐Richardson, Rebeka Silva, Chih‐Ko Yeh. Kevin J. Malley, Sam E. Farish, Mary B. Duffy, and Robert M. Craig contributed to data acquisition and critically revised the manuscript. All authors gave final approval and agreed to be accountable for all aspects of the work.

## CONFLICT OF INTEREST

The authors declare no conflict of interest.

## Data Availability

The data that support the findings of this study are available from the corresponding author upon reasonable request.

## APPENDIX A

**Table A1:** Oral white and red mucosal lesions studied with defining characteristics (adapted from [Westat, Inc, 1992])

1. Smokeless Tobacco‐associated Lesion
a) Mucosal lesions associated with smokeless tobacco use b) Classification based on texture and color c) Degree 1
i. Slight superficial wrinkling of mucosa‐wrinkles are fine, superficial, close together ii. Subtle color changes‐may range from normal to pale white/gray iii. Does not appear to be thickened, disappears when tissue is stretched
d) Degree 2
i) More defined wrinkling of mucosa‐wrinkles are more distinct and linear, wrinkling may disappear when tissue is stretched but color change remains ii) Distinct white/gray or occasional red color change iii) No thickening of mucosa
e) Degree 3
i) Distinct white/gray or occasional red color change ii) Broad bands of color change separated by furrows of normal or reddish color iii) Obvious thickening of mucosa iv) Wrinkling and furrows are evident even with stretching
2. Leukoplakia
a) White patch or plaque that cannot be wiped off b) Lesion cannot be characterized clinically as any other disease c) Appearance varies from a small, circumscribed area to extensive lesions involving a large area of the mucosa d) Surface appearance is variable: may be smooth or wrinkled, smooth‐surfaced but traversed with small cracks or fissures, or nodular/speckled (white areas intermingled with red areas) e) Color may be white, whitish‐yellow or gray f) Recommended subdivision:
1) Homogeneous—lesion is uniformly white with a smooth or corrugated surface 2) Nonhomogeneous—lesion in which a part is white and a part appears reddened
g) Three types of nonhomogeneous leukoplakia have been described:
1) Erythroleukoplakia (erosive leukoplakia)—white lesion that includes red areas 2) Nodular leukoplakia—lesion with slightly raised white areas (granules or nodules) interspersed with red areas 3) Verrucous leukoplakia—exophytic lesion with irregular sharp or blunt projections
3. Lichen Planus
a) Lesions show a great variation in clinical appearance b) All types may show the presence of Wickham's striae—delicate white lines of keratinized epithelium c) Lesions cannot be wiped off d) Clinical presentation includes:
1) Reticular form, with interlacing striae forming a lattice or annular pattern (most common type) 2) White, pinhead‐sized papules 3) White, plaque‐like lesions with striae at the margins 4) Erythematous area with striae at the margins 5) Atrophy of tongue papillae, with dry, whitish surface 6) Erosive or ulcerated areas with striae at the margins 7) Vesicles or bullae with clear or slightly red fluid associated with any of the above
4. Erythroplakia
a) Red patches or plaques that cannot be characterized clinically as any other condition
i. Color can be dull or bright ii. Surface texture is velvety or cobblestoned
5. Kaposi's sarcoma
a) Found in immunocompromised patients:
i. HIV‐positive, AIDS ii. Organ transplant recipients iii. Cancer chemotherapy or radiotherapy iv. Other types of immunosuppression
b) Intraoral macular lesions of red, purple, or blue hue c) Early lesions may resemble physiologic pigmentation d) Long‐standing lesions may be nodular and cover large areas of the palate and gingiva e) Lesions may progress rapidly f) Usually asymptomatic but may have painful ulcerations g) May present with skin lesions
6. Hairy leukoplakia
a) Found in immunosuppressed patients b) White corrugated hyperkeratotic projections on the lateral border of the tongue c) Lesion may be small or extensive, unilateral or bilateral, may appear on other mucosal sites (rare) d) May have superimposed candidal infection usually asymptomatic
7. Papilloma/verrucus vulgaris
a) Exophytic growth, usually pedunculated b) Verrucous, “cauliflower‐like” surface c) White, gray, or normal color is characteristic d) Found on the labial mucosa, palate, floor of the mouth
8. Nonspecific ulcer

**Table A2a:** Cigarette smoking and smokeless tobacco use by type of prevalent lesion at enrollment

	Smokeless tobacco lesions		Leukoplakia		Lichen planus		Virus‐associated lesions		Total^b^	
Tobacco use^a^	#	%	#	%	#	%	#	%	#	% (*p* Value)
Cigarette smoking										
Current user	1	5.0	15	55.6	2	16.7	6	42.9	25	33.3
Former user	9	45.0	10	37.0	9	75.0	4	28.6	33	44.0
Never user	10	50.0	2	7.4	1	8.3	4	28.6	17	22.7 (0.0004)
Cigarette pack‐years										
Noncigarette smoker	10	50.0	2	7.4	1	8.3	4	28.6	17	22.7
1–19	2	10.0	8	29.6	7	58.3	4	28.6	22	29.3
20–39	2	10.0	7	25.9	2	16.7	2	14.3	13	17.3
40–59	2	10.0	6	22.2	1	8.3	1	7.1	11	14.7
60 or more	4	20.0	4	14.8	1	8.3	3	21.4	12	16.0 (0.04)
Chewing tobacco and snuff use										
Current user	16	80.0	2	7.4	0	0	0	0	18	24.0
Former user	4	20.0	6	22.2	2	16.7	2	14.3	14	18.7
Never user	0	0	19	70.4	10	83.3	12	85.7	43	57.3 (0.0000)

^a^ Never user defined for cigarette smoking as having never smoked or smoked fewer than 100 cigarettes in a lifetime. Never user of chewing tobacco or snuff defined as having never used or used for less than 6 months.

^b^ Includes one participant with erythroplakia and one with unspecified ulcer.

**Table A2b:** Alcohol use by type of prevalent lesion at enrollment

	Smokeless tobacco lesions		Leukoplakia		Lichen planus		Virus‐associated lesions		Total^b^	
Alcohol use^a^	#	%	#	%	#	%	#	%	#	% (*p* Value)
Current drinker	8	40.0	11	40.7	7	58.3	6	42.9	32	42.7
Former drinker	11	55.0	16	59.3	5	41.7	6	42.9	40	53.3
Never drinker	1	5.0	0	0	0	0	2	14.3	3	4.0 (0.35)

^a^ Never drinker defined as having never consumed alcohol of any kind or had fewer than 12 drinks in a lifetime.

^b^ Includes one participant with erythroplakia and one with unspecified ulcer.
